# p-STAT3 is a PDC-E2 interacting partner in human cholangiocytes and hepatocytes with potential pathobiological implications

**DOI:** 10.1038/s41598-021-01060-5

**Published:** 2021-11-04

**Authors:** Ewa Kilanczyk, Jesus M. Banales, Ewelina Jurewicz, Piotr Milkiewicz, Malgorzata Milkiewicz

**Affiliations:** 1 Department of Medical Biology, Pomeranian Medical University, Szczecin, Poland; 2 Department of Liver and Gastrointestinal Diseases, Biodonostia Health Research Institute – Donostia University Hospital – Ikerbasque, CIBERehd, San Sebastian, Spain; 3 Nencki Institute of Experimental Biology, Warsaw, Poland; 4 Translational Medicine Group, Pomeranian Medical University, Szczecin, Poland; 5 Liver and Internal Medicine Unit, Medical University of Warsaw, Warsaw, Poland

**Keywords:** Biochemistry, Molecular medicine

## Abstract

The E2 component of the mitochondrial pyruvate dehydrogenase complex (PDC) is the key autoantigen in primary biliary cholangitis (PBC) and STAT3 is an inflammatory modulator that participates in the pathogenesis of many liver diseases. This study investigated whether PDC-E2 interacts with STAT3 in human cholangiocytes (NHC) and hepatocytes (Hep-G2) under cholestatic conditions induced by glyco-chenodeoxycholic acid (GCDC). GCDC induced PDC-E2 expression in the cytoplasmic and nuclear fraction of NHC, whereas in Hep-G2 cells PDC-E2 expression was induced only in the cytoplasmic fraction. GCDC-treatment stimulated phosphorylation of STAT3 in the cytoplasmic fraction of NHC. siRNA-mediated gene silencing of PDC-E2 reduced the expression of pY-STAT3 in NHC but not in HepG2 cells. Immunoprecipitation and a proximity ligation assay clearly demonstrated that GCDC enhanced pY-STAT3 binding to PDC-E2 in the nuclear and cytoplasmic fraction of NHC cells. Staining with Mitotracker revealed mitochondrial co-localization of PDC-E2/pS-STAT3 complexes in NHC and Hep-G2 cells. In cirrhotic PBC livers the higher expression of both PDC-E2 and pY-STAT3 was observed. The immunoblot analysis demonstrated the occurrence of double bands of PDC-E2 protein in control livers, which was associated with a lower expression of pY-STAT3. Our data indicate the interaction between PDC-E2 and phosphorylated STAT3 under cholestatic conditions, which may play a role in the development of PBC.

## Introduction

Pyruvate dehydrogenase complex (PDC) is a mitochondrial matrix multisubunit protein that converts pyruvate to acetyl-CoA. Recent studies indicated that PDC is present in the nucleus and processes/catalyzes histone acetylation^1–3^. In turn, the E2 component of PDC (PDC-E2) is the key autoantigen in primary biliary cholangitis (PBC), a chronic cholestatic liver disease with autoimmune phenome targeting intrahepatic bile duct lining cells, i.e., cholangiocytes^4^.

Aberrant overexpression of PDC-E2 in PBC cholangiocytes results in the production of anti-mitochondrial antibodies (AMA), which initiates the autoimmune response. Cholangiocytes are morphologically and functionally heterogeneous and impact other cells in the liver through inflammatory and fibrotic mediators, such as TNFα and IL-6^5^. Additionally, cholangiocytes are involved in cell-cycle phenomena that maintain tissue homeostasis in the biliary system via modulators of proliferation and apoptosis^6^.

When bile ducts are damaged, bile and other toxins build up in the liver (cholestasis) and slowly damage the liver tissue. Bile acid overload enhances a cascade of damage in hepatocytes, including mitochondrial membrane leakage, and leads to necrosis, apoptosis, and hepatocyte cell death^7^. Sustained cholestasis, in combination with the autoimmune reaction against cholangiocytes, leads to inflammation, cholangiocyte death, fibrosis, cirrhosis, and finally to liver failure^8^.

Signal transducer and activator of transcription factor 3 (STAT3) plays a critical role in the cellular response to a variety of cytokines and growth factors by regulating specific nuclear genes^9^. STAT3 is an inflammatory modulator that participates in the pathogenesis of liver diseases^10^. In contrast to the previously well documented hepatoprotective effect of STAT3^11–13^, this molecule plays a more complex role in liver inflammation. STAT3 can act either as a pro- or anti-inflammatory signal^10^. Recently, it has been demonstrated that a small pool of STAT3 (mitoSTAT3, 10–15%) is present in the mitochondria and enhances the activity of the electron transport chain (ETC)^14–18^. The nuclear translocation of STAT3 requires both tyrosine 705 (Y705) and serine 727 (S727) phosphorylation^19,20^. In turn, the acetylation and serine phosphorylation of STAT3 can restrict its mitochondrial translocation^21^.

As STAT3 is an inflammatory modulator that participates in the pathogenesis of liver diseases, and PDC-E2 is the key autoantigen in PBC, the aim of this work was to study whether these two proteins associate/interact in cholangiocytes and hepatocytes, and investigate how the pattern of their expression changes in cirrhotic/PBC derived tissues.

## Materials and methods

### Cell culture and immunoprecipitation (IP)

Primary normal human cholangiocytes (NHC) were cultured, as previously described^22^. Human hepatocytes (Hep-G2) were purchased from American Type Culture Collection (ATCC, Manassas, VA). Hep-G2 cells were grown in Eagle’s Minimum Essential Medium containing 10% fetal bovine serum (FBS; Gibco, Waltham, MA) and 100 U/mL penicillin, 100 µg/mL streptomycin (Sigma-Aldrich, St Louis, MO), and the cultures were maintained in the presence of 5% CO_2_ at 37 °C.

NHC and Hep-G2 cells were incubated with 100 µM or 250 µM of glycochenodeoxycholate (GCDC) acid, respectively. After 24 h, cellular fractionation was performed using the NE-PER™ Nuclear and Cytoplasmic Extraction Kit (Thermo Scientific™, #PI78833) according to the manufacturer’s procedure. Briefly, cells were harvested with trypsin–EDTA and then centrifuged at 1000 ×*g* for 5 min. After washing with PBS, ice-cold CERI buffer was added to the dry cell pellet, and incubation was carried out on ice for 10 min. Then, ice-cold CER II buffer was added, and the incubation was prolonged on ice for an additional 1 min.

After centrifugation (5 min at 14,000 ×*g*), the supernatant (cytoplasmic fraction) was transferred to a new tube while the pellet fraction containing the nuclei was incubated with ice-cold NER buffer. The samples were placed on ice and vortexed for 15 s every 10 min for a total of 40 min. After centrifugation (10 min at 14,000 ×*g*), the supernatant (nuclear fraction) was collected and transferred to new tubes. To measure the protein concentration in both the cytoplasmic and nuclear fractions, a bicinchoninic acid assay (Micro BCA™ Protein Assay Kit; Thermo Scientific) was performed.

For immunoprecipitation, 500 µl of the cytoplasmic and nuclear fractions containing 500 µg of proteins were incubated with 5 µg of antibody against PDC-E2 (Santa Cruz Biotechnology, #271534) or STAT3 (Sigma, #0255) overnight on a rotator at 4 °C. Protein A/G plus-agarose beads (Santa Cruz Biotechnology, #2003) were added to the antibody/epitope mixture, and the conjugation reaction proceeded on a rotator for an additional 2 h at 4 °C. Then, the beads were pelleted by centrifugation at 13,000 ×*g* for 5 min at 4 °C.

The supernatant (unbound fraction) was removed, and the pellet was washed three times with ice-cold IP buffer that contained 10 mM Tris (pH 7,4), 150 mM NaCl, 1 mM EGTA, 1 mM EDTA, 1% Triton X-100, and protease and phosphatase inhibitors). After the final wash, the IP antibody and immunoprecipitated proteins were eluted from the resin into SDS loading buffer (LB) by boiling the samples for 10 min at 95 °C. The samples were briefly centrifuged, and the supernatants were subjected to SDS-PAGE and western blotting. A mock sample containing no antibody was used as a negative control for each IP experiment (data not shown).

### Proximity ligation assay (PLA)

To visualize the PDC-E2/p-STAT3 complexes in normal human cholangiocytes (NHC) and in hepatocytes (Hep-G2), a proximity ligation assay (PLA; In situ PLA Technology, Olink Bioscience, Sigma-Aldrich, St Louis, MO) was applied. NHC and Hep-G2 cells were incubated with 100 µM and 250 µM GCDC, respectively, and were then fixed with 3% (w/v) paraformaldehyde in buffer containing 60 mM PIPES, 25 mM HEPES, pH 6.9, 5 mM EGTA, and 4 mM MgCl_2_ for 20 min at RT.

After washing with this buffer, the cells were permeabilized for 4 min with 0.1% (v/v) Triton X-100 at 4 °C and washed again. All the following steps were performed according to the manufacturer’s protocol using the reagents (except primary antibodies) and buffers provided in the PLA kit. The reaction included rabbit polyclonal anti-PDC-E2 (#LS-C665101) and mouse monoclonal anti-p-STAT3 (Y705; Cell Signaling, #4113) antibodies diluted 1:500 and was carried out for 1.5 h at RT.

After washing, the incubation with anti-rabbit PLA plus and anti-mouse PLA minus probes (1:4) was conducted for 1 h at 37 °C in a humidity chamber. After the ligation and amplification steps, the coverslips were immobilized on the glass slides with the mounting medium containing DAPI. In the control experiments, the cells were treated as above but without the addition of ligase. The cells were analyzed under a Zeiss LSM 780 confocal microscope (Munich, Germany) using a 63×/1.4 oil immersion objective lens.

### Preparation of liver tissue lysates

Control liver tissues (n = 13) were obtained from large margin liver resections of colorectal metastases. PBC liver tissues with histologically proven cirrhosis were collected during liver transplantations for PBC patients (n = 15). Non-cirrhotic (early-stages (F0 and F2) of PBC) liver species were obtained by percutaneous needle liver biopsy (2–3 mm^2^) from PBC patients. Alcoholic liver disease (ALD) liver tissues with histologically proven cirrhosis were collected during liver transplantation. The total lysate from the control and PBC/cirrhotic liver tissues was prepared as described previously^23^.

### SDS-PAGE and western blot

Proteins (40 µg) from the lysates were obtained from the liver tissues or the total and fractionated cells (NHC or Hep-G2) and were run on SDS polyacrylamide gels and then blotted onto PVDF membranes (Thermo Scientific) under semi-dry transfer conditions. The membranes were incubated in Tris-buffered saline Tween-20 (TBS-T) containing 5% milk for 1 h at RT. The membranes were probed with the following primary antibodies: anti-PDC-E2 (Santa Cruz, #271534; 1:500 dilution), anti-p-STAT3 (Y705; Cell Signaling, #4113; 1:500 dilution) anti-p-AKT (Cell Signaling, #9271; 1:500 dilution), anti-GAPDH (Santa Cruz, #47724; 1:5000 dilution), and anti-HDAC-2 (Santa Cruz, #81599; 1:1000 dilution), and were then incubated overnight at 4 °C.

Afterward, the membranes were washed and incubated for 2 h at RT with anti-rabbit (Amersham ECL, Rabbit IgG #NA9340) or anti-mouse (Johnson ImmunoResearch, #115-035-146) HRP-conjugated secondary antibodies diluted at 1:5000 in TBS-T with 5% milk. Protein bands were detected with a chemiluminescence detection system (Millipore) and visualized and quantified with the MicroChemi 2.0 System and GelQuant software (Israel).

### Immunohistochemistry

The localization and level of PDC-E2 and p-STAT3 were examined in frozen liver sections (6 µm) using immunohistochemistry. Briefly, frozen sections were fixed with acetone at -20 °C for 5 min. Then, they were incubated with 3% H_2_O_2_ followed by exposure to the Avidin/Biotin blocking kit (Vector Laboratories, #SP-2001). After blocking with 10% NHS (Normal Horse Serum) for 30 min, the sections were probed with primary antibodies: anti-PDC-E2 (Santa Cruz, #271534, diluted 1:250) and p-STAT3 (Y705; Cell Signaling, #4113, diluted 1:100) for 24 h at 4 °C. Next, biotinylated anti-mouse/anti-rabbit IgGs (#BA-1400, Vector Laboratories) were used as secondary antibodies. The reactions were visualized using the ABC Vectastain and DAB kit (Dako, Agilent Technologies, Denmark). Additional counterstaining with Mayer’s Hematoxylin staining (DAKO) was also performed. The negative control, in which the primary antibodies were omitted, was included in the study and uniformly demonstrated no reaction. To confirm the membrane localization of PDC-E2, an immunofluorescent assay was performed as described previously^24^. Images were acquired with a ZEISS Axio Imager Z2 fluorescence microscope.

### Immunofluorescence analysis with MitoTracker

To check whether PDC-E2/p-STAT3 interaction occurs in mitochondria, NHC and Hep-G2 cells were treated with 100 µM and 250 µM GCDC, respectively. Then cells were incubated with 500 nM MitoTracker-Deep Red FM (#8778, Cell Signaling) for 30 min at 37 °C and 5% CO_2_ followed by fixation, permeabilization and blocking processes as we described previously for PLA staining. Incubation with PDC-E2 (Santa Cruz, #271534) and p-STAT3 (S727) (Abcam, #30647) antibodies diluted 1:500 were carried out overnight at 4 °C. After washing with PBS, the incubation with secondary donkey anti-rabbit antibody conjugated with FITC (#711-095-152 Jackson ImmunoResearch, 1:500 dilution) and anti-mouse antibody conjugated with Rhodamine (#T15-295-150 Jackson ImmunoResearch, 1:500 dilution) was conducted for 1 h at RT. Then cells were mounted on slides using the Vectashield mounting medium with DAPI (#H-1200, Vector Laboratories, Burlingame, USA) to visualized cell nuclei and analyzed using Olympus FV 1000 confocal system with IX81 inverted microscope.

### Small interfering RNA (siRNA)transfection

NHC and Hep-G2 cells were transfected with PDC-E2 or scrambled siRNAs (10 nM per 1 × 10^5^ cells; #SR301209, Origen, Rockville, MD, USA) using Lipofectamine RNAiMAX (Thermo Fisher Scientific, Waltham, MA, USA) according to the manufacturer’s instructions. Cells in six-well plates at 60% confluency were incubated for 48 h to achieve a maximum gene silencing.

### Statistical analysis

The statistical significance of all data was analyzed using StatView software version 5 (SAS Institute Inc., Carry, NC, USA). Significant differences were determined using Fisher’s PLSD test; *p* < 0.05 was accepted as significant.

### Ethics

Written informed consent was obtained from each patient included in the study. The study protocol was approved by the Ethics Committee of Pomeranian Medical University and conforms to the ethical guidelines of the 1975 Declaration of Helsinki (6th revision, 2008).

## Results

### PDC-E2 associates with p-STAT3 or STAT3 in cholangiocytes and hepatocytes mainly after response to GCDC

**Figure 1:**
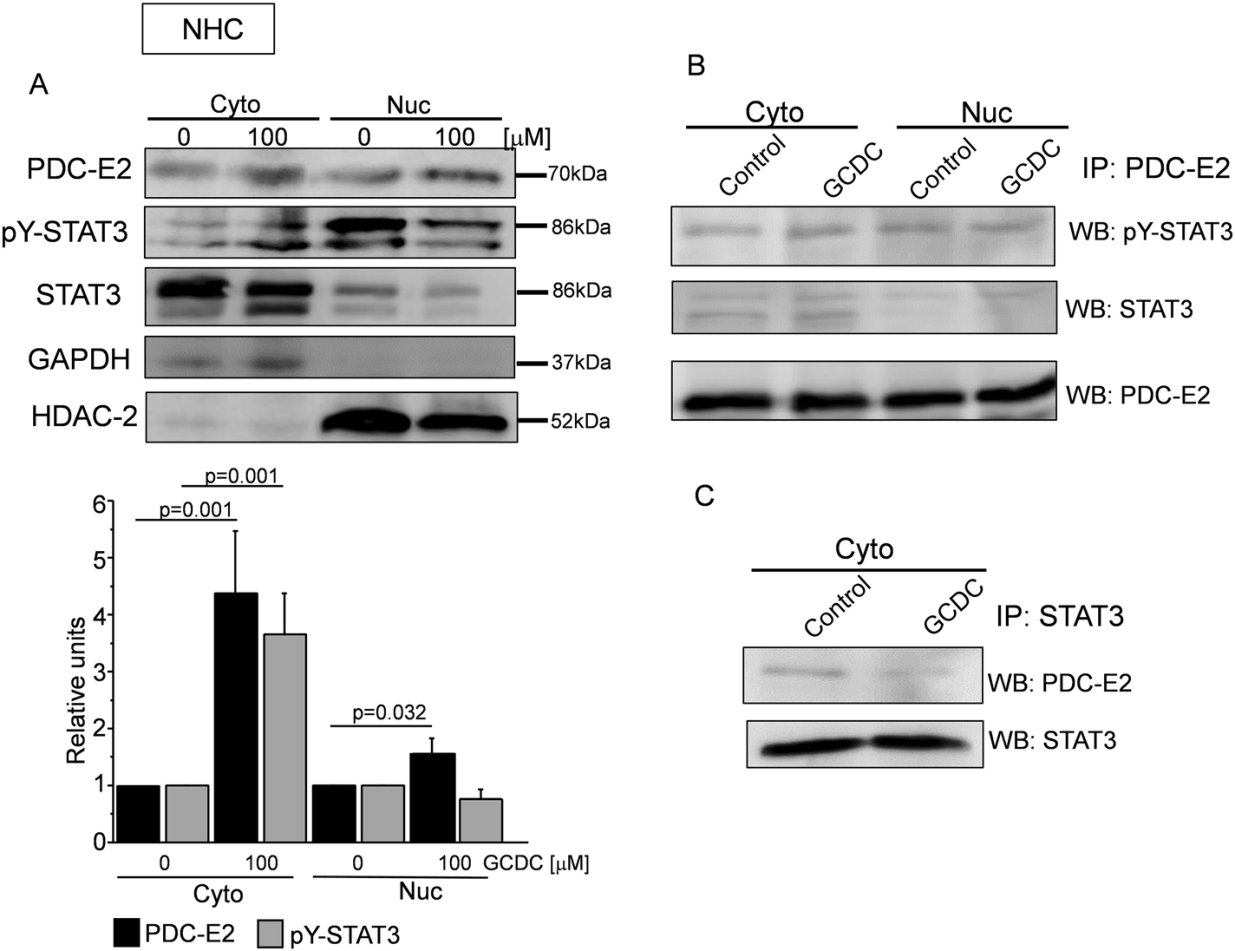
PDC-E2 associates with p-STAT3 and STAT3 in unstimulated and GCDC-stimulated cholangiocytes. Normal human cholangiocytes (NHC) stimulated with 100 µM of glycochenodeoksycholic acid (GCDC) were collected and subjected to subcellular fractionation in order to obtain cytoplasmic (Cyto) and nuclear (Nuc) fractions. (**A**) PDC-E2, pY-STAT3 and STAT3 were detected by using appropriate antibodies and normalized to GAPDH (cytoplasmic fraction) or HDAC-2 (nuclear fraction). Level of each protein was presented as a mean ± SEM. (**B** and **C**) Immunoprecipitation (IP) utilized anti-PDC-E2 or anti-STAT3 antibody and contained 500 µg proteins per reaction. PDC-E2 (70 kDa) and pY-STAT3 or STAT3 (both 86 kDa) were detected by Western blot (WB). IP experiments were repeated three times on independent batches of cells.

To determine whether STAT3 associates with PDC-E2 in cholangiocytes (NHC) and hepatocytes (HEp-G2) the immunoprecipitation (IP) assay was performed. The cells were incubated with 100 µM (NHC) and 250 µM (Hep-G2) GCDC, and, 24 h later, the cytoplasmic and nuclear fractions were obtained. We found that in NHC cells, GCDC increased the level of PDC-E2 and p-STAT3 (Y705) in the cytoplasmic fraction by 4.3-fold and 3.6-fold vs. the control (both *p* = 0.001) and the level of PDC-E2 in the nuclear fraction by 1.5-fold vs. the control (*p* = 0.032) (Fig. 1A).

IP performed with antibodies against PDC-E2 followed by western blotting developed with anti-pY-STAT3 or anti-STAT3, showed an association of PDC-E2 with pY-STAT3 in both the cytoplasmic and nuclear fraction of NHC cells stimulated, or not, with GCDC, whereas an association of PDC-E2 with STAT3 presented mostly in the cytoplasmic fraction of cells treated with GCDC (Fig. 1B). In turn, IP with antibodies against STAT3 indicated that the interaction between PDC-E2 and STAT3 was present in the cytoplasmic fraction of the NHC cells both non-stimulated and stimulated with GCDC (Fig. 1C).

**Figure 2:**
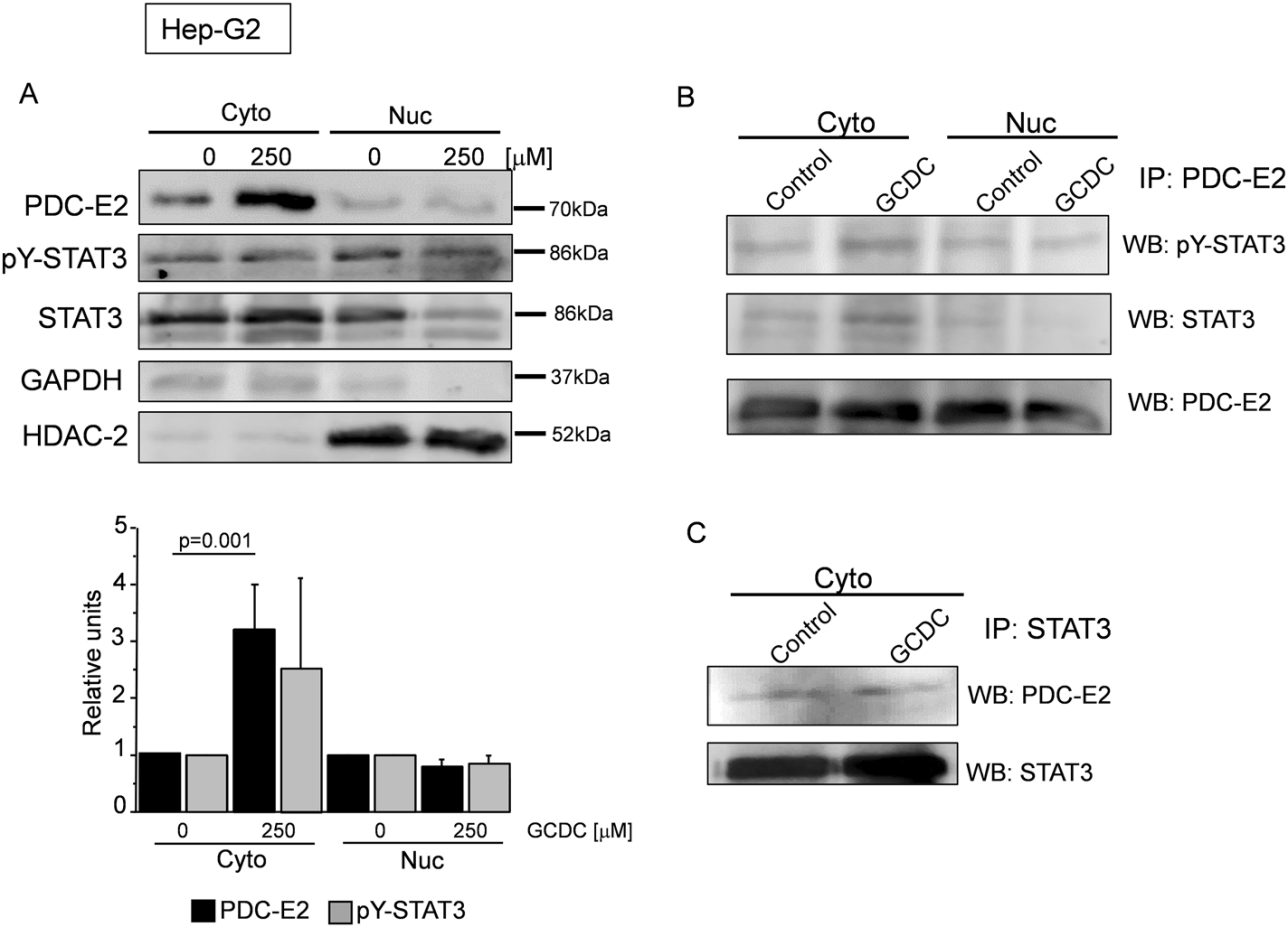
PDC-E2 associates with p-STAT3 and STAT3 in GCDC-stimulated hepatocytes. Hepatocytes (Hep-G2) stimulated with 250 µM of glycochenodeoksycholic acid (GCDC) were collected and subjected to subcellular fractionation in order to obtain cytoplasmic (Cyto) and nuclear (Nuc) fractions. (**A**) PDC-E2, pY-STAT3 and STAT3 were detected by using appropriate antibodies and normalized to GAPDH (cytoplasmic fraction) or HDAC-2 (nuclear fraction). Level of each protein was presented as a mean ± SEM. (**B** and **C**) Immunoprecipitation reaction (IP) utilized anti-PDC-E2 or anti-STAT3 antibody and contained 500 µg proteins per reaction. PDC-E2 (70 kDa) and pY-STAT3 or STAT3 (both 86 kDa) were detected by Western blot (WB). IP experiments were repeated three times on independent batches of cells.

In the Hep-G2 cells, GCDC elevated the level of PDC-E2 (3.2 fold vs. the control; *p* = 0.001) whereas it did not influence the level of pY-STAT3 in the cytoplasmic fraction (Fig. 2A). Regarding the nuclear fraction, no statistically significant changes were observed for PDC-E2 and pY-STAT3 (Fig. 2A). IP performed with anti-PDC-E2 antibodies revealed an association of PDC-E2 with pY-STAT3 and STAT3 in the cytoplasmic fraction of Hep-G2 cells after exposure to GCDC (Fig. 2B). An association of PDC-E2 with STAT3 proteins in the cytoplasmic fraction was proved using an IP assay with antibodies against STAT3 (Fig. 2C).

**Figure 3:**
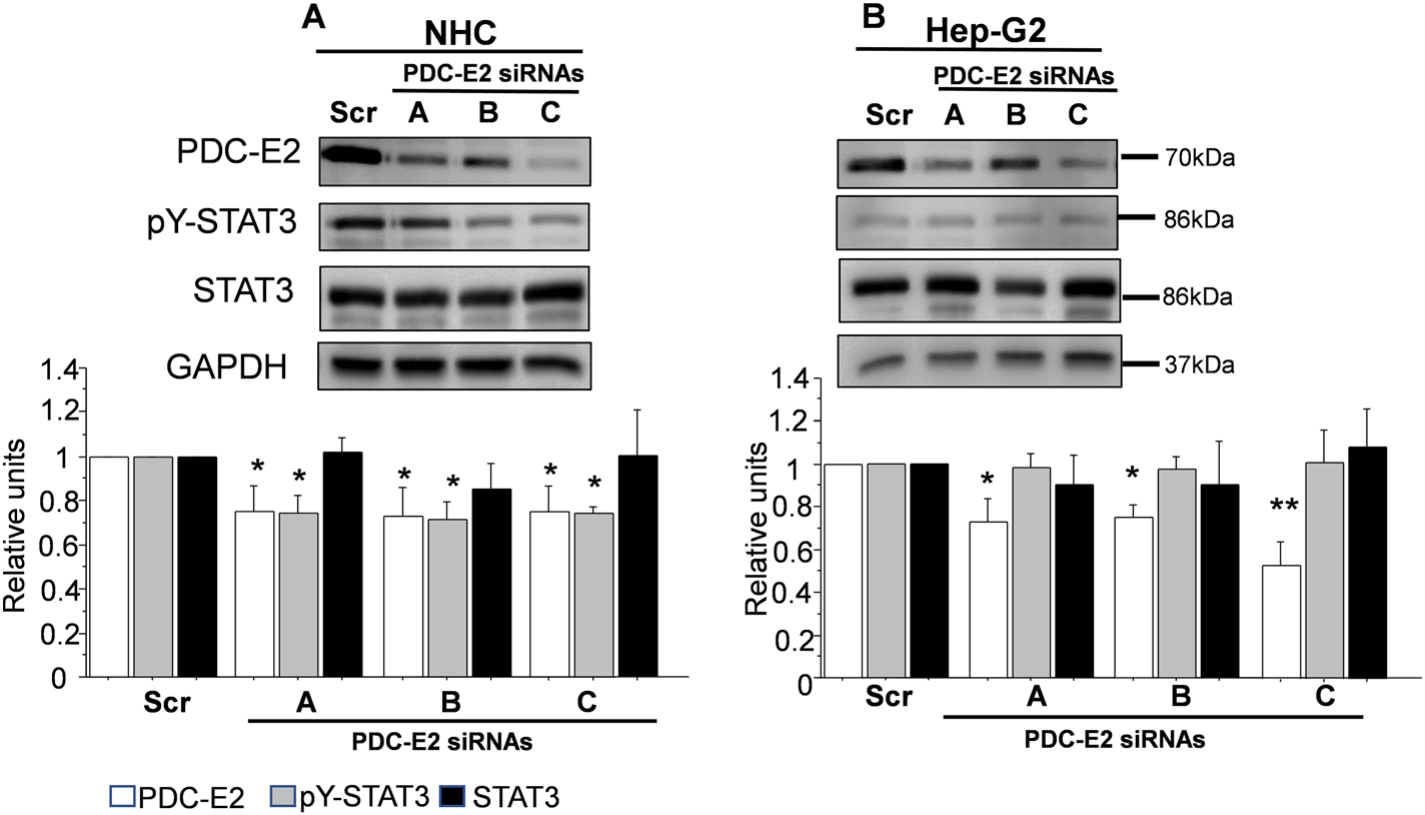
Gene silencing of PDC-E2 reduces p-STAT3 level in NHC cells. Western blot analysis of PDC-E2, pY-STAT3 and STAT3 protein levels in NHC **(A)** and Hep-G2 **(B**) cells treated with three siRNAs (**A**, **B**, **C**) to silence PDC-E2 or a scrambled (scr) siRNA. Results are representative of three independent experiments. **p < 0.01, *p < 0.05.

To evaluate if PDC-E2 influences the phosphorylation of STAT3 we used an siRNA approach, targeting PDC-E2 mRNA. A nonspecific scrambled RNA served as a control. All used siRNAs significantly lowered PDC-E2 level in both NHC and Hep-G2 cells (Fig. 3A,B) but only in NHC cells the level of pY-STAT3 was reduced by the respective PDC-E2 siRNAs (30% reduction for each siRNA, *p* < 0.05) (Fig. 3A).

**Figure 4:**
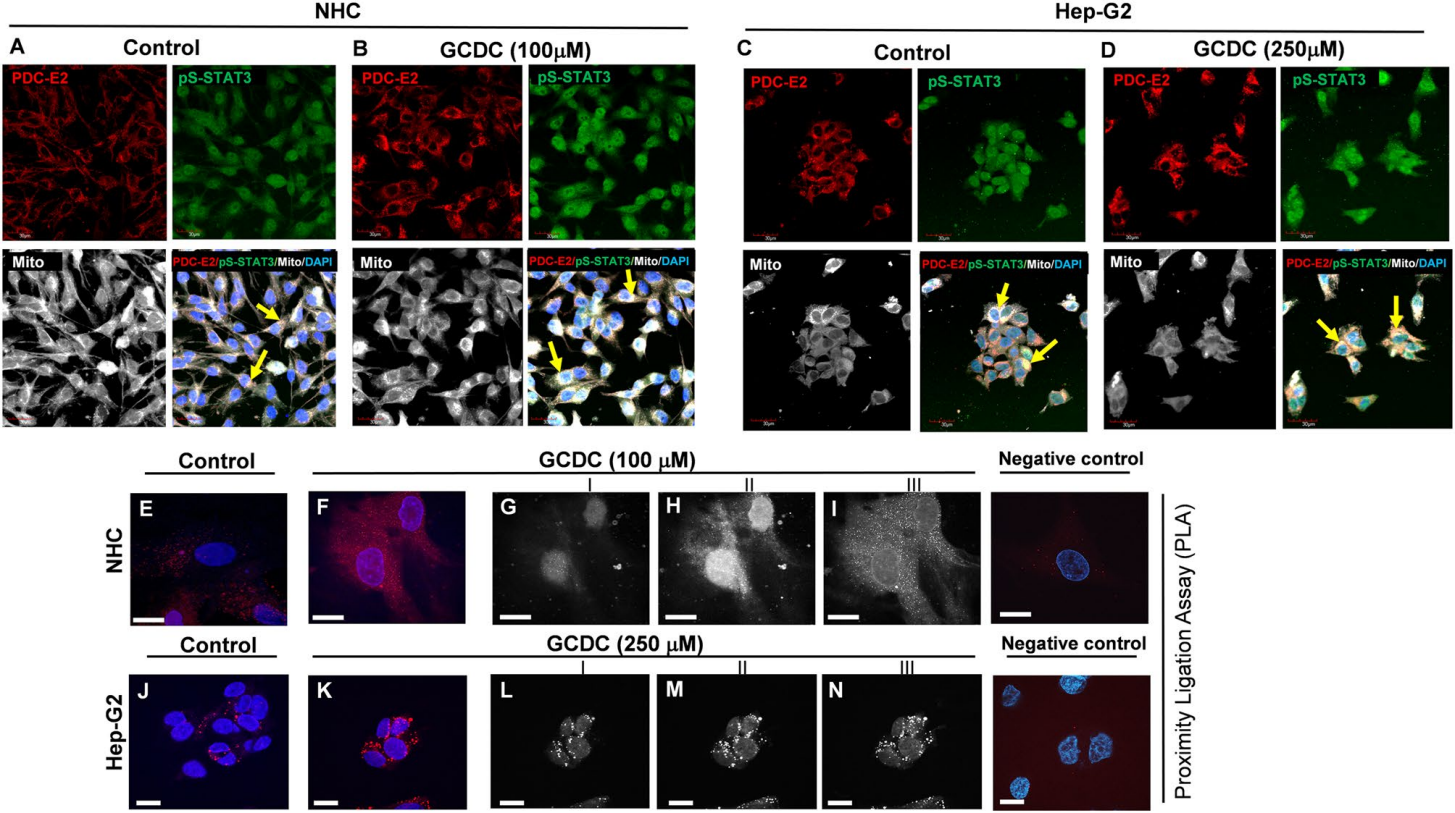
The presence of PDC-E2—p-STAT3 complexes in human cholangiocytes (NHC) and hepatocytes (Hep-G2). Human cholangiocytes (NHC) and hepatocytes (Hep-G2) were incubated with 100 or 250 µM of GCDC, respectively. In order to show mitochondrial localization of PDC-E2/pS-STAT3 complexes cells were incubated with 500 nM of MitoTracker. Immunofluorescence analysis revealed co-localization of PDC-E2 and p-STAT3 (S727) with MitoTracker in NHC (**A** and **B**) and Hep-G2 cells (**C** and **D**) (yellow arrows). Objective magnification ×20, zoom 3. PLA in situ hybridization assay was conducted using primary antibodies against PDC-E2 and p-STAT3 (Y705). PLA signals (red dots) indicate for the interaction between PDC-E2 and p-STAT3 in both NHC (**E** and **F**) and HepG2 (**J** and **K**) cells. Nuclear (**G** and **H**) and cytoplasmic (**I**) localization of PDC-E2-pY-STAT3 complexes in NHC cells and cytosolic localization of PDC-E2-pY-STAT3 complexes in Hep-G2 cells (**L–N**) are shown using three different z-stack. Scale bar is 30 µm. Nuclei were counterstained with DAPI. This experiment was repeated two times on independent batches of cells.

To check whether the interaction between PDC-E2 and p-STAT3 (S727) occurs in mitochondria of cholangiocytes (NHC) and hepatocytes (Hep-G2), cells were treated with 100 µM and 250 µM of GCDC, respectively followed by incubation which MitoTracker probes, which accumulate in active mitochondria. Immunofluorescent assay revealed that PDC-E3 co-localized with pS-STAT3 within NHC and Hep-G2 mitochondria (Fig. 4A,C, yellow arrows). The interaction is more evident in cells treated with GCDC (Fig. 4B,D, yellow arrows).

In order to further explore the idea that the PDC-E2 interacts directly with pY-STAT3 we employed a proximity ligation assay (PLA), which is an advanced immunofluorescence technique that allows visualizing stable and transient interactions of proteins in different cellular compartments. The PLA assay revealed the formation of PDC-E2/pY-STAT3 complexes (fluorescence signal visualized as red dots), in both NHC (Fig. 4E) and Hep-G2 (Fig. 4J) cells. In the presence of an elevated level of GCDC, the formation of these complexes were observed in both cytoplasm and nuclei of NHC cells (Fig. 4F) whereas in Hep-G2 cells mostly in cytoplasm (Fig. 4K). To more precisely visualize the nuclear localization of PDC-E2/pY-STAT3 complexes, different z-stacks from NHC (Fig. 4G–I) and Hep-G2 (Fig. 4L–N) cells are presented.

### The localization and level of PDC-E2 and pY-STAT3 in cirrhotic/PBC liver tissues

**Figure 5:**
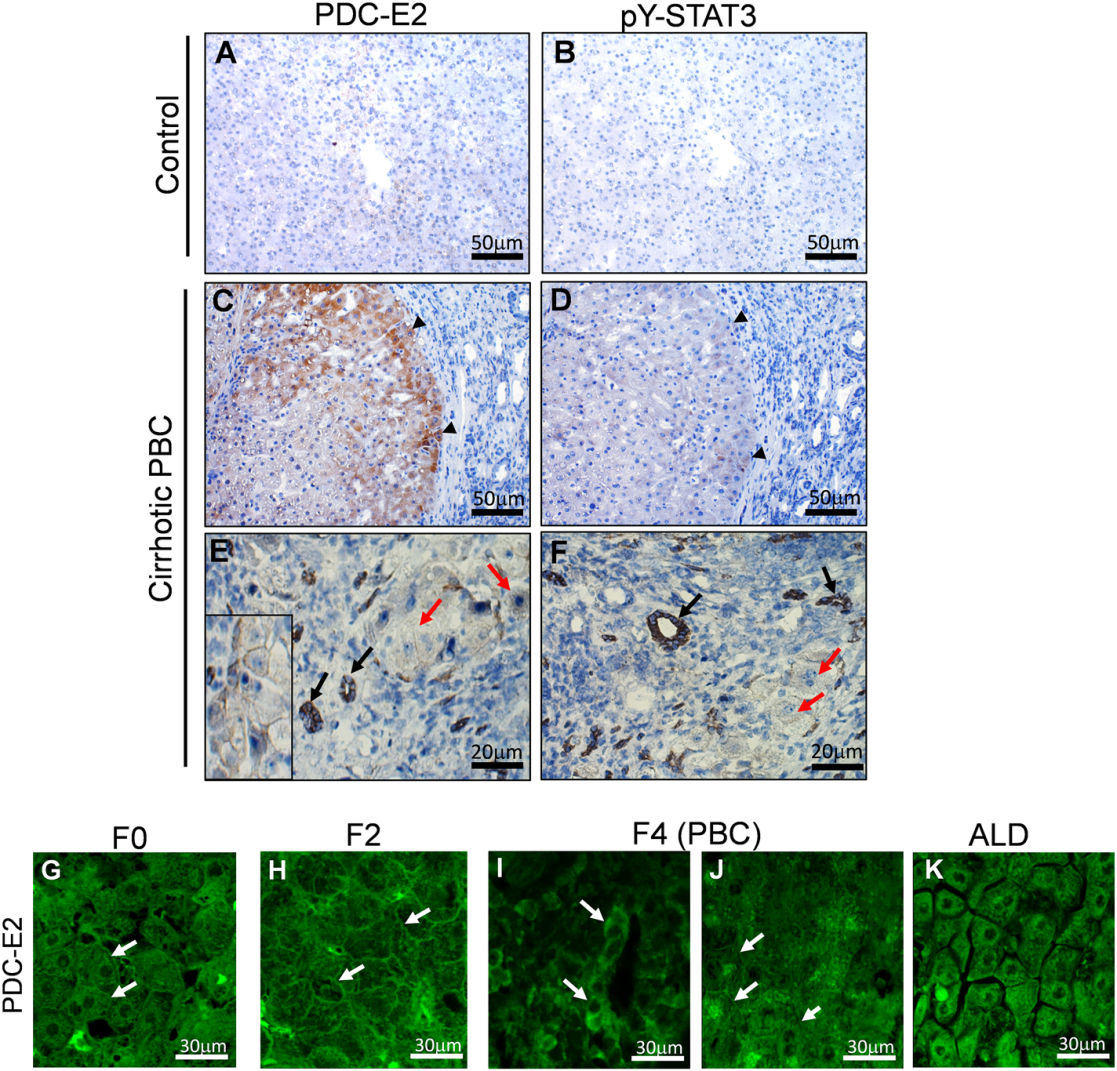
Localization of PDC-E2 and p-STAT3 in cirrhotic (PBC or ALD) and control liver tissues. Immunohistochemical analysis shows that in cirrhotic/PBC tissues, PDC-E2 (**C**) and pY-STAT3 (**D**) are present mainly on the edge of regenerating nodules in contrast to control tissues, in which both examined proteins are barely visible (**A** and **B**). Additionally, in PBC tissues PDC-E2 and pY-STAT3 are present in both cholangiocytes (**E** and **F**, red arrows) and hepatocytes (**E** and **F**, black arrows). Scale bar—50 and 20 µm. A representative immunofluorescent staining of PDC-E2 at different stages of PBC development (**G**–**J**) and of ALD tissues (**K**) are shown. Membrane localization of PDC-E2 is pointed by white arrows. Scale bar is 30 µm. Objective magnification ×20, zoom 3.

In the next step, we analyzed the localization and level of PDC-E2 and pY-STAT3 in the control and cirrhotic PBC livers. In cirrhotic livers (Fig. 5C,D), immunohistochemical staining for both examined proteins was stronger than in the controls (Fig. 5A,B) and was limited to regenerative hepatic nodules (black arrowheads). The expression of PDC-E2 (Fig. 5E) and pY-STAT3 (Fig. 5F) was present in both cholangiocytes (black arrows) and hepatocytes (red arrows). In cirrhotic livers, PDC-E2 was present in the cellular membrane (Fig. 5E, insert).

The fluorescent detection of PDC-E2 shows its membrane localization at the early-pre-cirrhotic F0, F2, stages of PBC (Fig. 5G, H respectively, white arrows). At the cirrhotic, F4 stage of PBC, PDC-E2 was present in the cellular membrane of both cholangiocytes (Fig. 5I) and hepatocytes (Fig. 5J). To confirm that membrane localization of PDC-E2 concerned only PBC, we performed staining on liver tissue with histological-proven cirrhosis, derived from alcoholic liver disease (ALD) patients. As can be seen in Fig. 5K, and as was expected, in this tissue PDC-E2 was localized mainly in the cytoplasm.

**Figure 6:**
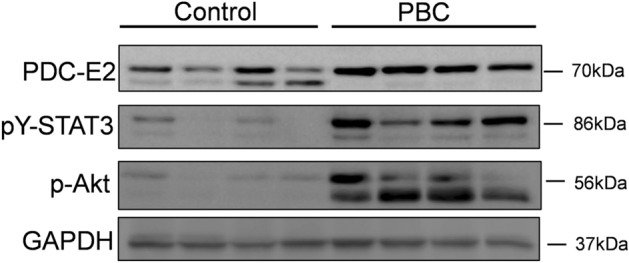
Presence of PDC-E2 and STAT3 in liver tissue samples derived from control and PBC patients. Immunoblot analysis of the liver tissue samples shows the presence of an additional (low-molecular weight band) of PDC-E2 protein corresponding to lower level of pY-STAT3 in control tissues whereas in cirrhotic/PBC liver tissues, PDC-E2 was detected as single band corresponding to higher level of pY-STAT3. Level of each proteins was normalized to GAPDH as a loading control.

Finally, when proteins from the liver tissues were analyzed by immunoblot, we found that antibodies against PDC-E2 recognized two bands in the control tissues and only a single band in the cirrhotic PBC livers. Since, in cirrhotic livers, the level of pY-STAT3 is higher than in the controls (Fig. 6), it seems that appearance of PDC-E2 double bands correlates with a lower level of pY-STAT3.

## Discussion

The current knowledge on PDC-E2 functioning as an autoantigen in the liver tissues of PBC patients is still limited and intensively investigated. Since STAT3 participates in the pathogenesis of liver diseases and exhibits both nuclear and mitochondrial activities^15,25–27^, we analyzed a new interaction between PDC-E2 and STAT3 in both the cytoplasmic and nuclear fractions of human cholangiocytes (NHC) and hepatocytes (Hep-G2) following incubation with a toxic concentration of bile salt (GCDC). This model mimics bile acid (BA) overload, which develops at a hepatic level in PBC due to insufficient trans-hepatocyte BA flow caused by reduced sinusoidal and/or canalicular BA transport^7^.

In our study, the co-immunoprecipitation experiment indicated that PDC-E2 interacted with both pY-STAT3 and STAT3 in control and GCDC-stimulated human cholangiocytes (NHC) and hepatocytes (Hep-G2). The similar PDC-E2-STAT3 interaction was reported previously but in different type of cells, namely adipocytes (3T3-L1)^28^. The interaction of PDC-E2 with another member of STAT family, STAT5 in adipocytes and adipose tissues or leukemic T cells was also established^28,29^. We further confirmed this physical association between PDC-E2 and STAT3 by proximity ligation assay, a tool which allows in situ detection of protein interactions with high specificity and sensitivity. To the best of our knowledge, this is the first demonstration of this interaction in cholangiocytes and hepatocytes.

Moreover, we demonstrated that GCDC enhanced the PDC-E2-STAT3 interaction. In cholangiocytes it is apparent in both the cytoplasm and nucleus, whereas in hepatocytes this enhanced interaction was mostly observed in the cytoplasm. The nuclear presence of PDC-E2 in NHC cells may suggest its involvement in either histone acetylation, as it was proven in lung cancer cells (A549)^1–3^ or in formation of DNA binding complex as it was observed in adipocytes^28,29^. Our observation that the association between STAT3 and PDC-E2 is more evident when STAT3 was phosphorylated may suggest that bile acid-GCDC may induce the phosphorylation of STAT3^30^. This may occur via Akt activation as cross-talk between Akt kinases and the JAK/STAT3 signaling pathway was reported in Hep-G2 cells^31^. In the current study, we found that the level of phospho-Akt was elevated in the PBC liver tissues. IL-6, which expression is enhanced in PBC livers, may also be involved in STAT3 phosphorylation as it is an upstream cytokine in the JAK/STAT3 pathway^30^.

To understand the functional aspect of PDC-E2/pY-STAT3 interaction in NHC and Hep-G2 cells we used the siRNA approach targeting PDC-E2. Knocking down the expression of PDC-E2 greatly reduces the phosphorylation of STAT3 (Y705) in NHC but not in Hep-G2 cells. This positive correlation between PDC-E2 and pY-STAT3, presented in cholangiocytes, the cells primary affected in PBC, may suggest the role of PDC-E2-modulated STAT3 phosphorylation during development of cholestasis.

Furthermore, since the observed STAT3-PDC-E2 interaction was also localized within the cytoplasm we further investigated if it is present within mitochondria. MitoSTAT3 was found to be associated with the inner mitochondrial membrane^32^. The immunofluorescent assay with MitoTracker and antibodies against PDC-E2 and pS-STAT3 (S727) clearly showed that PDC-E2/pS-STAT3 complexes are co-localized within mitochondria in both cholangiocytes (NHC) and hepatocytes (Hep-G2). MitoSTAT3 is not required for mitochondrial formation but its phosphorylation at serine727 (pS-STAT3) enhances the ETC complex activity^15^. Though not yet clear, there is some evidence that while mitoSTAT3 positively regulates ETC activities and increases mitochondrial membrane potential its mitochondrial localization is associated with the increased ROS production. Our study showed that GCDC can induce the phosphorylation of mitoSTAT3 what may affect the ROS production.

In accordance with our in vitro studies, the immunohistochemistry analysis showed a more intense staining for PDC-E2 and pY-STAT3 in cirrhotic livers of PBC patients in comparison to the control tissue, and those proteins were localized mainly in cholangiocytes within bile ducts. Additionally, the increased staining of PDC-E2 was noticed within cytoplasm/membrane of hepatocytes. The immunofluorescent detection of PDC-E2 revealed membrane expression of this protein in hepatocytes, which was observed as early as at the pre-cirrhotic stage 0 (F0) and stage 2 (F2) of PBC, suggesting its presence outside the mitochondria before the cirrhosis expanded. This membrane localization of PDC-E2 could shed a light on its autoantigen action during PBC development. This phenomenon appears to be specific to PBC, where this protein is abnormally distributed and overexpressed, as this localization is not seen in other cirrhotic conditions, such as alcohol-induced liver cirrhosis in ALD patients.

Finally, in liver extracts, immunoblot analysis revealed that the PDC-E2 protein was manifested as two bands. The extra low-molecular-weight band may be related to another isoform of the protein and this occurrence correlates with the lower level of p-STAT3. This low-molecular weight band might be an alternatively spliced PDC-E2 isoform presented mainly in healthy liver tissues. Of note, this extra band of PDC-E2 protein was also observed in the control NHC cells during the IP experiment. The lack of this band in hepatocytes suggests that the extra band of PDC-E2 in liver tissues mostly reflects the pattern of PDC-E2 in cholangiocytes.

There are some important limitations of this study, including the lack of an alternative splicing analysis of PDC-E2, which needs to be performed using the mass spectroscopy method to prove the presence of a new isoform of this protein. In our opinion however, presented data, despite limitations, is important also in the context of using biological material (liver biopsies) from patients suffering from PBC. This material is less available nowadays for research work since according to current guidelines liver biopsy is no longer necessary to establish a diagnosis of PBC in patients with positive AMA antibodies and elevated alkaline phosphatase. Also non-invasive methods (i.e., elastography) are becoming more commonly used for the diagnosis of liver fibrosis.

In summary, our results indicated that the PDC-E2 association with p-STAT3 under cholestatic conditions may significantly impact its cellular function and may play a role in the pathogenesis of PBC.

## Supplementary Information

Supplementary Information.

## Abbreviations

DTT: Dithiothreitol
GCDC: Glyco-chenodeoxycholic acid
PAGE: Polyacrylamide gel electrophoresis
PBS: Phosphate buffer saline
PLA: Proximity ligation assay
PBC: Primary biliary cholangitis
PDC: Pyruvate dehydrogenase complex
SDS: Sodium dodecyl sulfate
STAT3: Signal transducer and activator of transcription factor 3
DAPI: 4′,6-Diamidino-2-phenylindole dihydrochloride
PIPES: Piperazine-N,N′-bis (2-ethanesulfonic acid)
HEPES: N-(2-Hydroxyethyl)piperazine-N′-(2-ethanesulfonic acid)
TBS: Tris buffer saline
TBS-T: Tris buffer saline containing Tween 20
ALD: Alcoholic liver disease
TNF: Tumor necrosis factor
