# Supplementary figures and images for: p-STAT3 is a PDC-E2 interacting partner in human cholangiocytes and hepatocytes with potential pathobiological implications
Source: Sci Rep. 2021 Nov 4;11:21649. doi: 10.1038/s41598-021-01060-5 (PMC8569217; doi:10.1038/s41598-021-01060-5)

Fig 1A

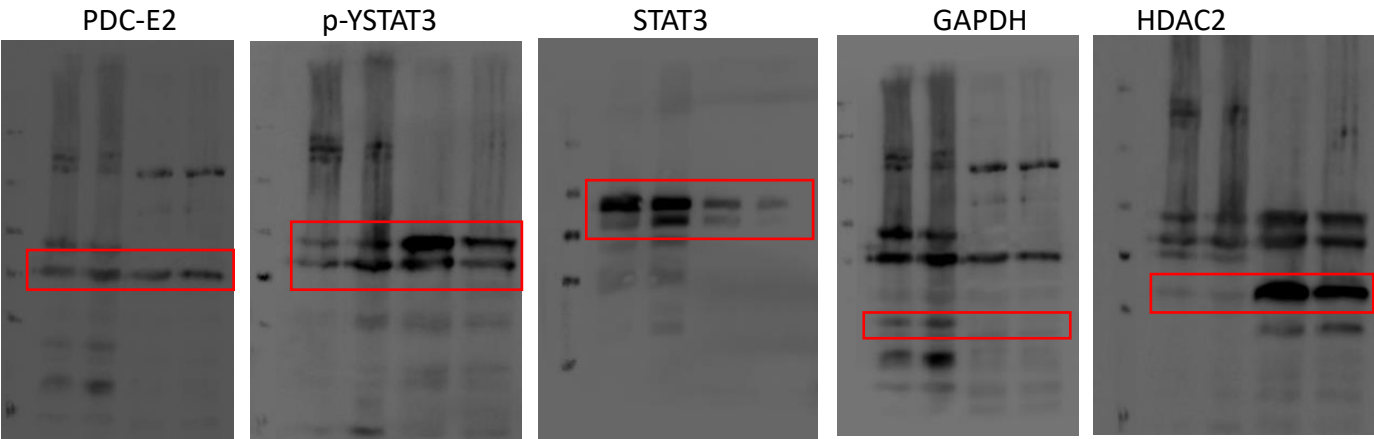

Fig 1B

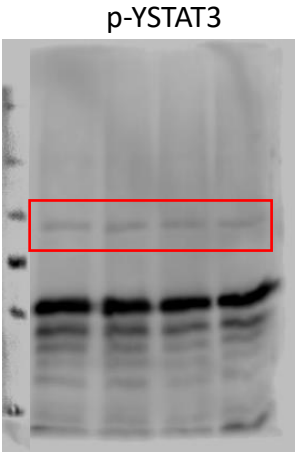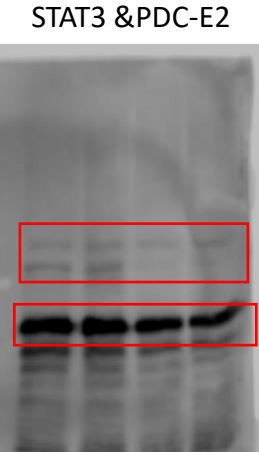

Fig 1C

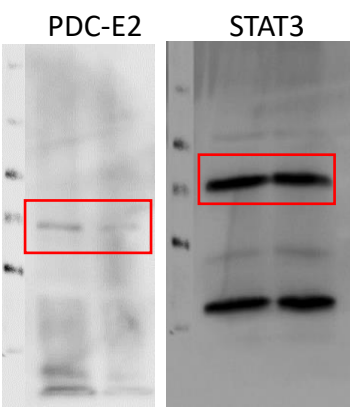

Fig 2A

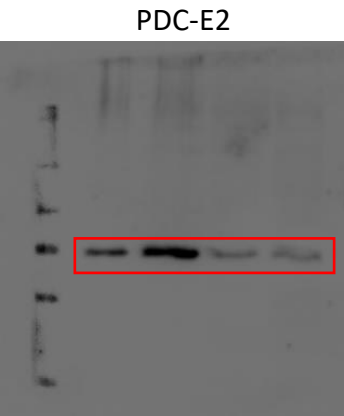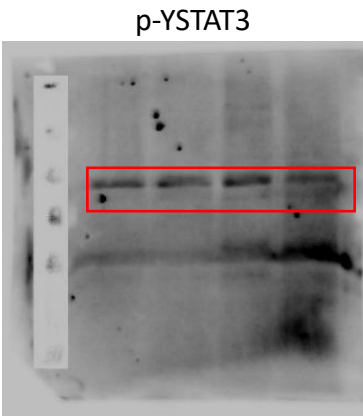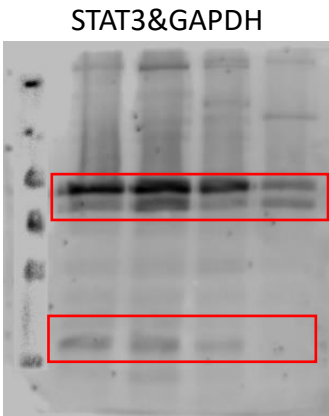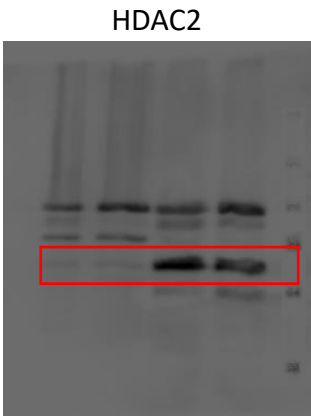

Fig 2B

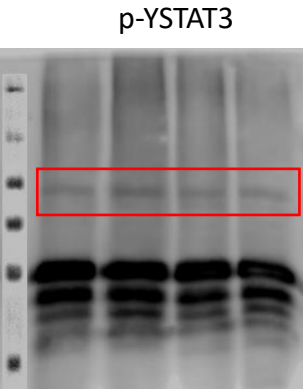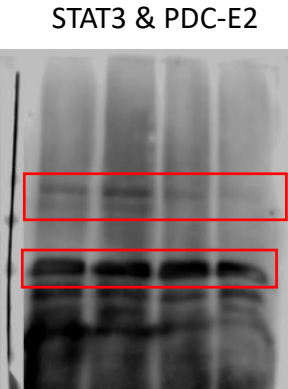

Fig 2C

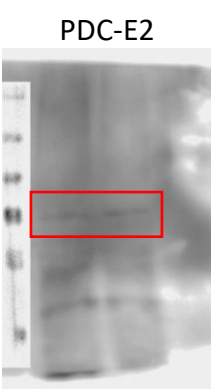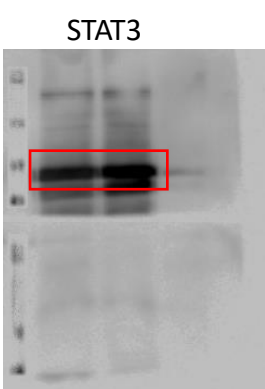

Fig 3

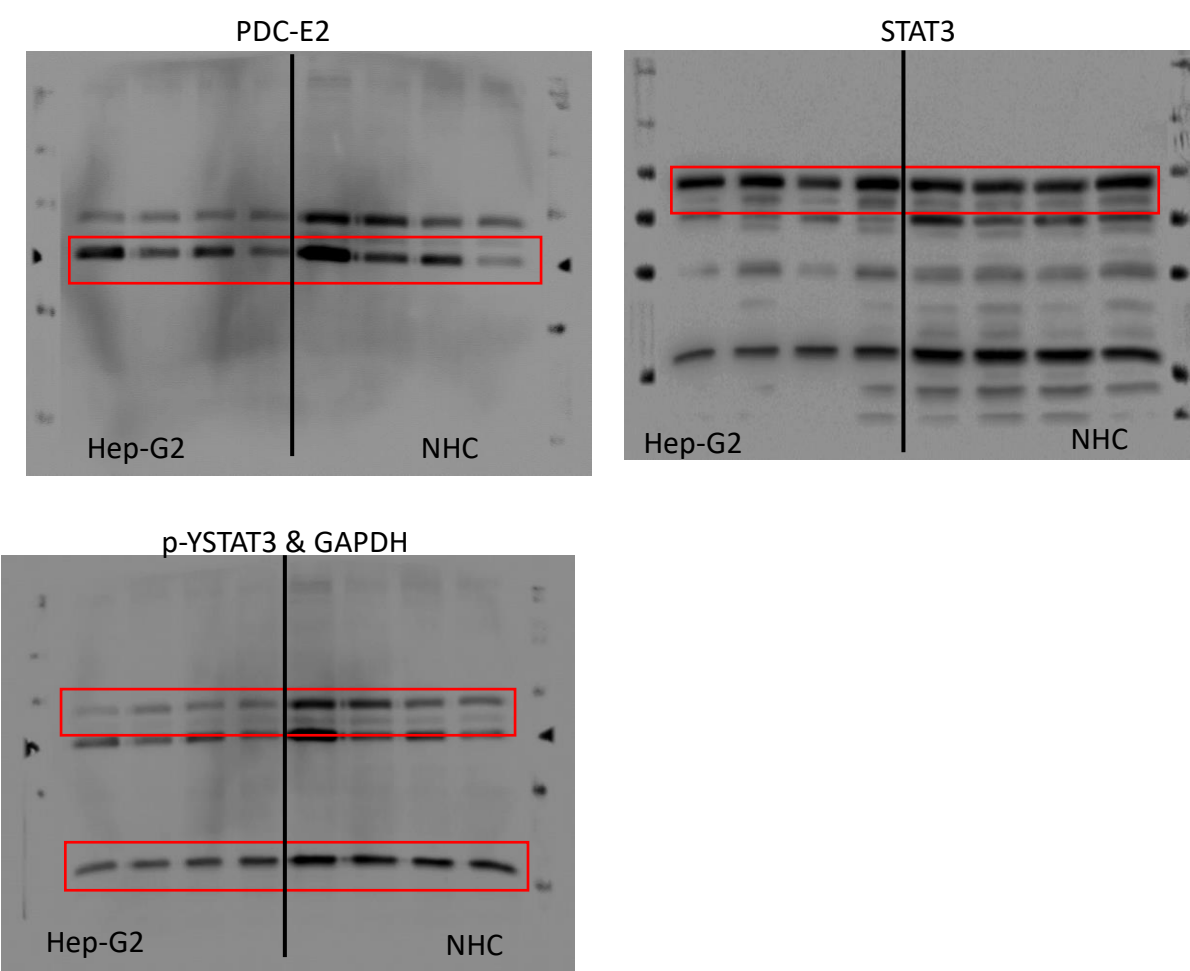

Fig 6

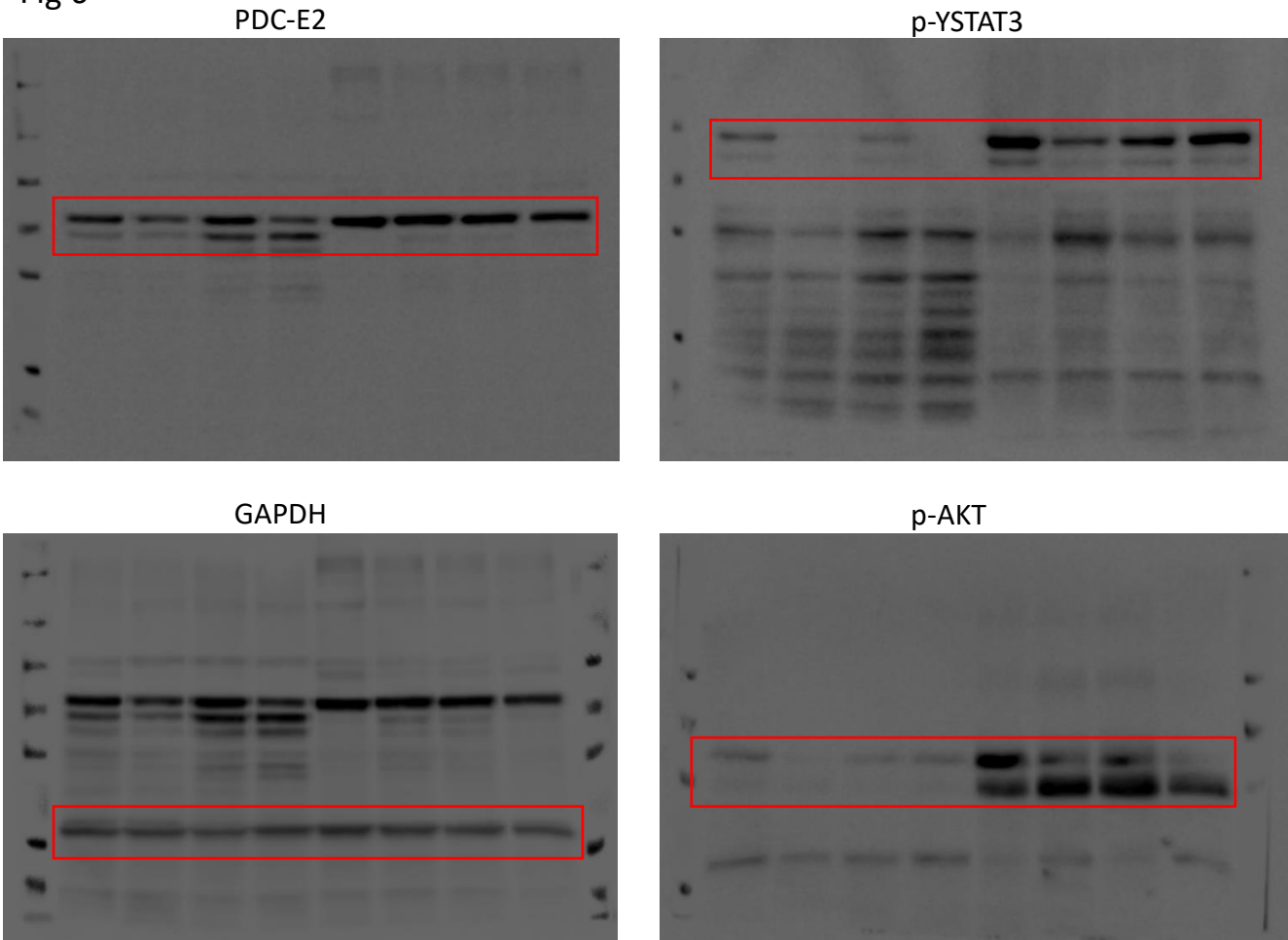

Supplement: Supplementary file 1 — Supplementary Information. [file 41598_2021_1060_MOESM1_ESM.pdf]
